# Long non‐coding RNA MEG3 knockdown attenuates endoplasmic reticulum stress‐mediated apoptosis by targeting p53 following myocardial infarction

**DOI:** 10.1111/jcmm.14714

**Published:** 2019-10-20

**Authors:** Xueling Li, Jinxuan Zhao, Jin Geng, Fu Chen, Zilun Wei, Chen Liu, Xinlin Zhang, Qiaoling Li, Jingmei Zhang, Ling Gao, Jun Xie, Biao Xu

**Affiliations:** ^1^ Department of Cardiology Zhejiang provincial People’s Hospital People’s Hospital of Hangzhou Medical College Hangzhou China; ^2^ Department of Cardiology Drum Tower Hospital Nanjing University Medical School Nanjing China; ^3^ Department of Cardiology Drum Tower Hospital Nanjing Medical University Nanjing China; ^4^ Department of Cardiology Yizheng Hospital Nanjing Drum Tower Hospital Group Yangzhou China

**Keywords:** cardiomyocyte apoptosis, endoplasmic reticulum stress, lncRNA MEG3, myocardial infarction, p53

## Abstract

Mounting evidence has indicated that long non‐coding RNA maternally expressed gene 3 (lncRNA MEG3) regulates cell apoptosis, and is involved in a variety of diseases. However, its exact role in myocardial infarction (MI) has not been fully elucidated. In the present study, we firstly observed that the expression levels of the lncRNA MEG3 in infarct hearts and hypoxic neonatal mice ventricular myocytes (NMVMs) were up‐regulated by quantitative real‐time PCR (qRT‐PCR). Then, we knocked down lncRNA MEG3 by lentiviral delivery in the myocardial border region following multipoint injection. Following 28 days of MI, the lncRNA MEG3 knockdown mice indicated better cardiac function, and less cardiac remodelling by ultrasonic cardiogram and histological analysis. In addition, we indicated that lncRNA MEG3 knockdown reduced myocyte apoptosis and reactive oxygen species production in MI mice model and hypoxic NMVMs. Furthermore, we revealed that knockdown of lncRNA MEG3 protected against endoplasmic reticulum stress (ERS)‐mediated myocardial apoptosis including the induction of PERK‐eIF2α and caspase 12 pathways. At last, we provided evidence that p53 was identified as a protein target of lncRNA MEG3 to regulate NF‐κB‐ and ERS‐associated apoptosis. Taken collectively, our findings demonstrated that lncRNA MEG3 knockdown exerted cardioprotection by reducing ERS‐mediated apoptosis through targeting p53 post‐MI.

## 1 INTRODUCTION

Myocardial infarction (MI) has become a major epidemic of cardiovascular diseases with high mortality across the world, despite the continuous development of new therapies.^1^, ^2^ It is generally known that cardiomyocyte apoptosis is the intersection for post‐infarction injury, leading to ventricular remodelling and heart failure.^3^, ^4^ Therefore, the strategy of attenuating MI‐induced cardiomyocyte apoptosis is always a significant target for myocardial repair.

Long non‐coding RNAs (lncRNAs) are non‐coding RNA larger than 200 nucleotides in length that have been recognized as important regulators in various cellular processes.^5^, ^6^ Several lncRNAs have been shown to regulate cardiomyocyte death following MI including lncRNA MIAT, CARL, MDRL and APF.^7^, ^8^ Maternally expressed gene 3 (MEG3) is located in human chromosome 14q32 at a region that approximately covers 1.6 kb nucleotides. This gene was found to act as a tumour suppressor and to possess highly conserved expression in multiple tissues.^9^, ^10^ The expression levels of lncRNA MEG3 were down‐regulated in various types of human tumours, contributing to increase cellular apoptosis via the regulation of p53 protein, microRNA‐127 and microRNA‐21‐5p.^11^, ^12^, ^13^ With regard to the cardiovascular system, the knockdown of lncRNA MEG3 could alleviate hypoxia‐induced H9c2 cell injury.^14^ However, the role and underlying mechanism of lncRNA MEG3 in MI remain unclear.

In the present study, we investigated the alterations in the expression levels of lncRNA MEG3 both in infarcted heart and hypoxic neonatal mice ventricular myocytes (NMVMs). Furthermore, we knocked down the expression levels of lncRNA MEG3 in mice heart tissues by lentiviral delivery in order to assess the role of lncRNA MEG3 in cardiomyocyte apoptosis and cardiac remodelling post‐MI. Lastly, the detailed mechanism of lncRNA MEG3 in cardiac repair following MI was elucidated in vivo and in vitro*.*

## 2 MATERIALS AND METHODS

### 2.1 Animal and myocardial infarction (MI) model

Male C57/BL6J mice (6‐8 weeks old) were obtained from the model animal research centre of the Nanjing University and were approved by the Institutional Ethics Committee of the Nanjing Drum Tower Hospital. The studies were performed according to the guidelines of US Department of Health (NIH Publication No. 85‐23, revised 1996) for use and care of laboratory animals and the conduct of primary cell culture.

The mice were anaesthetized with ketamine hydrochloride (50 mg/kg) and diazepam (2.5 mg/kg), subjected to operation of myocardial infarction model by ligation of the left anterior descending coronary artery (LAD) and killed as described previously.^15^ To investigate the expression of lncRNA MEG3 in the infarct zone of heart after MI, animals were randomly divided into two groups as follows: MI and sham. The infarct zones of the hearts were collected at 2, 4, 6, 12, 24 hours, and 1 week after surgery (n = 7 each time‐point per group). In order to evaluate the effects of lncRNA MEG3 knockdown on infarcted heart, 70 mice were assigned to three groups. The sham group mice were injected by an intramyocardial administration route with DPBS (n = 20), whereas the other two groups were treated with lentiviruses containing si‐MEG3 (n = 25) and/or GFP (n = 25) in the border zone immediately after LAD ligation. The viruses were administered in a total of 15 µL (1.5 × 10^7^ viral particles) by an intramyocardial route into three regions of the border zone using a 29‐gauge Hamilton syringe in mice undergoing myocardial infarction. The sham group mice were treated with open thoracotomy and intramyocardial injection of equal amount of DPBS. Ultrasonic cardiogram (UCG) was performed on surviving animals prior to and at periods of 1 and 4 weeks following surgery. The hearts of the animals were collected for further analysis including Western blotting, qRT‐PCR and immunohistochemistry. The detailed descriptions of the assays are provided in the online‐only Data S1.

### 2.2 Isolation of primary neonatal mice ventricular myocytes (NMVMs) and cell culture

Newborn ICR mice (1‐2 days old) were anaesthetized and killed, and their heart ventricles were rapidly and finely homogenized and digested in trypsin‐EDTA 0.125% (Invitrogen) at 4°C overnight. Collagenase (Invitrogen, 1 mg/mL in DMEM) was used to further digest the tissues in shanking bath at 37°C within 10 minutes as described previously.^15^ Subsequently, the supernatants were collected and centrifuged at 200 g for 5 minutes and the cells were cultured in DMEM containing 1 g/L glucose plus 10% FBS and 1% penicillin/streptomycin (GIBCO) at 37°C After 1.5 hours of incubation, the cells were diluted to a density of 1 × 10^6^ cells/mL and plated in 10 µg/mL laminin‐coated different culture dishes according to the experimental requirements. The purity of isolated NMVMs was estimated to be higher than 95% (>95%) based on the results of the α‐actin staining (Figure S1A). Anoxia was performed as described in previous studies.^16^ Briefly, the cells were placed in an anoxic chamber with a water‐saturated atmosphere composed of 5% CO_2_ and 95% N_2_. The hypoxic NMVMs were collected at 1, 4, 6, 12 and 24 hours (n = 3 each time‐point per group). The detailed methods for lentiviral transfection and the determinations of cell apoptosis and ERS are available in Data S1.

### 2.3 Statistical analysis

Data were presented as mean ± SD at least three independent experiments. The differences in data were analysed by unpaired, two‐tailed Student's *t* test for two groups or one‐way analysis of variance (ANOVA) for multiple comparisons. SPSS 22.0 (IBM SPSS) was used for statistical analyses, and statistical significance was considered as *P* < .05.

## 3 RESULTS

### 3.1 Dynamic changes in lncRNA MEG3 in infarct hearts and hypoxic cardiomyocytes

**Figure 1:**
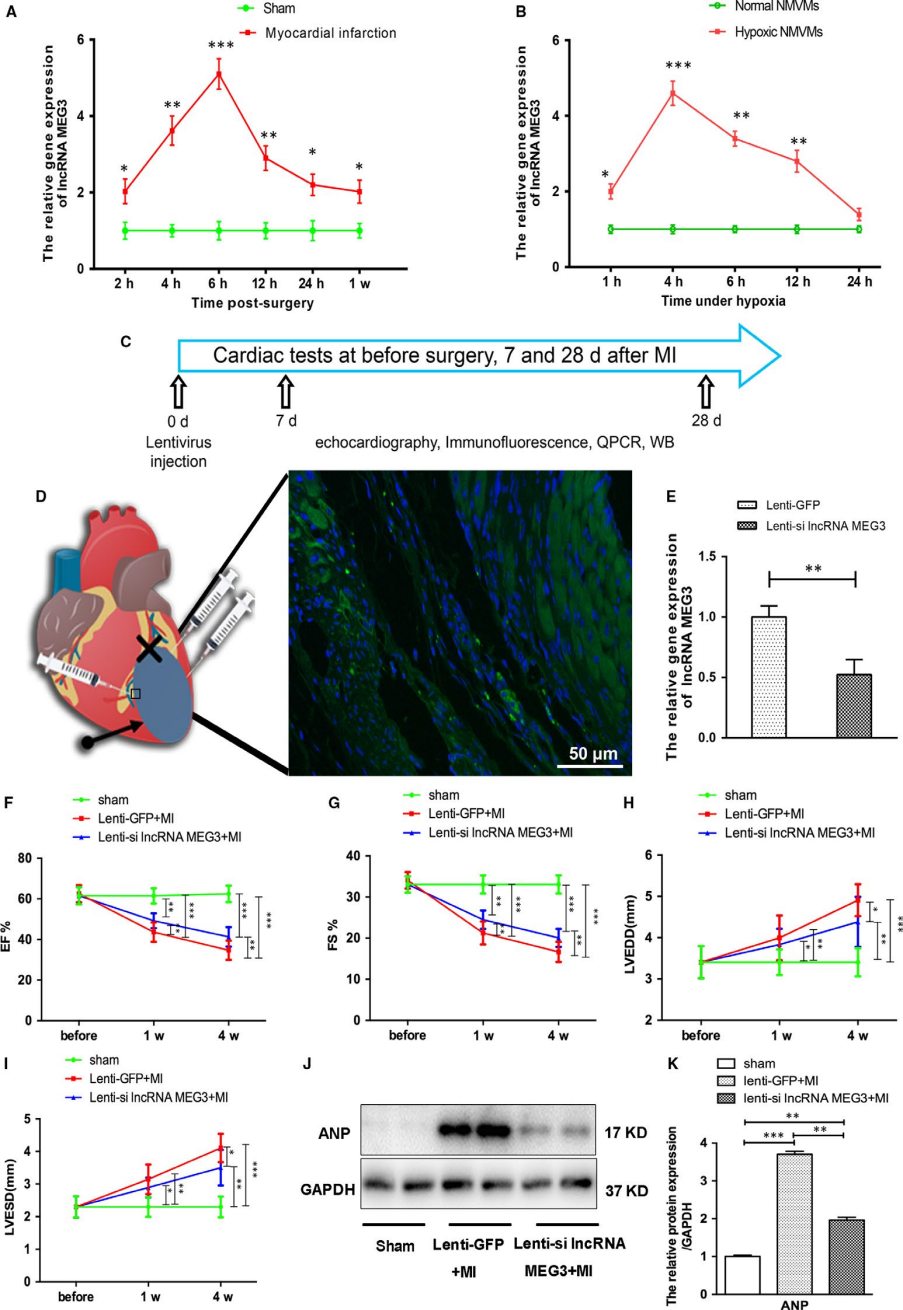
Expression profile of lncRNA MEG3 in infarct hearts and neonatal mice ventricular myocytes (NMVMs) under hypoxia. A, The lncRNA MEG3 expression levels in the infarct hearts were assessed by qRT‐PCR at the indicated time‐points following surgery (n = 7 each time‐point per group). The expression levels of lncRNA MEG3 expression were normalized to those of GAPDH expression and were expressed as fold change compared with the sham group. B, NMVMs were exposed to hypoxia, and lncRNA MEG3 expression levels in NMVMs were analysed at different time‐points (n = 3 each time‐point per group). C, Schematic representation of the animal experimental design. D, Schematic representation of lentiviral injection. The arrow indicates the infarcted area. The fork represents the ligation site of the left anterior descending artery (LAD). Immunofluorescence stains of GFP distribution at day 28 following MI. Scale bar = 50 μm. E, The qRT‐PCR analysis of lncRNA MEG3 expression around the injection site at 4 wk following MI (n = 7 each group). (F‐I) EF, FS, LVEDD and LVESD of mice from the three groups at 1 and 4 wk post‐MI. J‐K, The protein levels of ANP were detected by immunoblotting and quantified in mice heart tissues at 4 wk post‐MI (n = 7 each group). Values expressed as mean ± SD; *P < .05, **P < .01, ***P < .001

To identify the potential effect of lncRNA MEG3 during myocardial infarction (MI), we measured its expression levels in infarct hearts and hypoxic neonatal mice ventricular myocytes (NMVMs) by quantitative real‐time PCR (qRT‐PCR). The expression levels of lncRNA MEG3 in post‐MI myocardium were significantly higher than those of sham group for the indicated time‐points. The expression levels of lncRNA MEG3 were increased in mice at 2 hours following MI and demonstrated a continuous increase to the highest‐point level at 6 hours. The levels were gradually decreased over the remaining time‐points (Figure 1A). Subsequently, we assessed the expression levels of lncRNA MEG3 in NMVMs under hypoxia. Increased levels of lncRNA MEG3 were noted under hypoxic conditions in NMVMs that ranged from 2.03 ± 0.19‐fold at 1 hour to 4.60 ± 0.32‐fold at 4 hours and then were recovered to 1.39 ± 0.16 times at 24 hours (Figure 1B). These results revealed the up‐regulation of lncRNA MEG3 in infarct hearts and hypoxic cardiomyocytes, indicating the pathophysiological role of lncRNA MEG3 in MI.

### 3.2 The effects of lncRNA MEG3 knockdown on cardiac function

To explore the role of lncRNA MEG3 in MI, we knocked down its expression levels in the heart tissues of the animals by lentiviral delivery. The viral particles of lncRNA MEG3 interference vectors (MEG3 shRNA‐1, MEG3 shRNA‐2) and of the control lentiviral vector (scramble NC) with green fluorescent proteins (GFP) were transfected into NMVMs for 72 hours. Immunofluorescence and flow cytometry analyses indicated that the lentiviral transfection efficiency in NMVMs reached 79.02 ± 3.53% (Figure S1B,C). qRT‐PCR indicated that the inhibitory efficiency noted in NMVMs in the presence of MEG3 shRNA‐1 was higher than that noted in the presence of MEG3 shRNA‐2 (Figure S1D). Therefore, lentiviral vectors with MEG3 shRNA‐1 (lenti‐si lncRNA MEG3) and control lentivirus (lenti‐GFP) were used for the animal studies. The GFP marker was observed in the border zone around the injection sites by immunofluorescence 28 days following lentivirus intramyocardial injection in heart (Figure 1C,D). Following lenti‐si lncRNA MEG3 treatment post‐MI, lncRNA MEG3 levels were significantly decreased to 52.35 ± 12.41%, indicating successful delivery and lncRNA MEG3 knockdown in the target tissue (Figure 1E).

To verify whether lncRNA MEG3 knockdown affected the cardiac function after MI, we performed UCG at 1 week and 4 weeks post‐LAD ligation. Following induction of MI, all mice exhibited significant functional impairment at 1 week and 4 weeks compared with the sham‐treated mice. The mice of the lenti‐si lncRNA MEG3 group displayed a significant improvement on left ventricle ejection fraction (EF) and fractional shortening (FS) at 1 week and 4 weeks compared with that of lenti‐GFP group (Figure 1F,G). Additional parameters including left ventricular end‐systolic diameter (LVESD) and end‐diastolic diameter (LVEDD) were decreased in the lenti‐si lncRNA MEG3–treated mice 4 weeks following surgery compared with the lenti‐GFP–treated mice (Figure 1H,I).

Atrial natriuretic peptide (ANP) is a biomarker used in heart failure that is closely related to the severity of ventricular remodelling.^17^, ^18^ Our preliminary analysis by UCG indicated improved cardiac function following lncRNA MEG3 siRNA treatment. In agreement with these findings, the protein levels of ANP in lenti‐si lncRNA MEG3–treated mice were markedly decreased compared with those noted in lenti‐GFP–treated mice at 4 weeks, though these protein levels were still higher than those of sham‐treated animals (Figure 1J,K). The data revealed that inhibition of lncRNA MEG3 expression could improve the cardiac function following MI.

### 3.3 The effects of lncRNA MEG3 knockdown on myocardial infarct size

**Figure 2:**
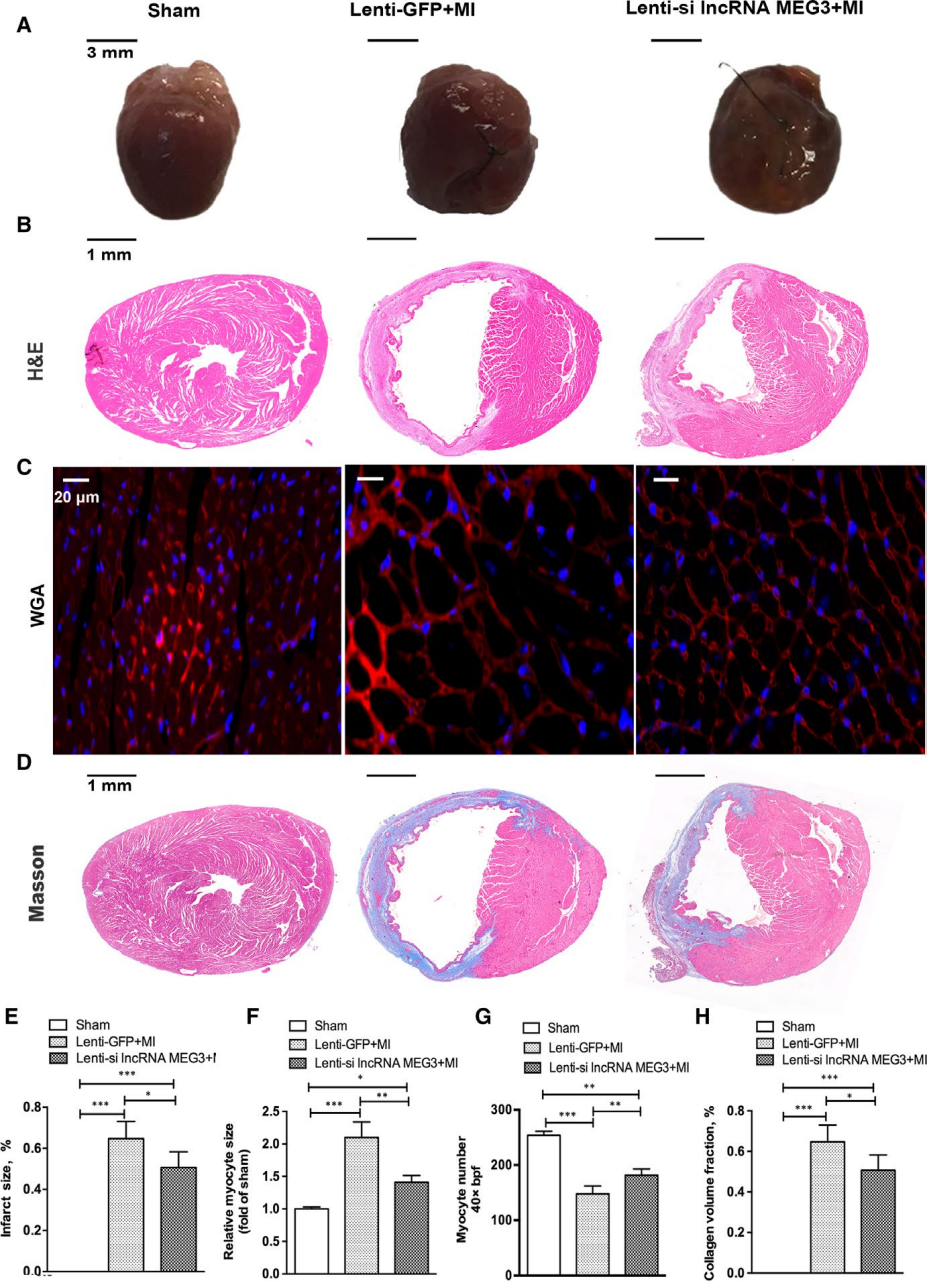
lncRNA MEG3 knockdown reduced infarcted size and cardiac fibrosis. Hearts were collected for analysis at 28 days post‐MI. A, Representative images and bar graph of heart tissues from sham, lenti‐GFP and lenti‐si lncRNA MEG3 mice were shown (n = 3 each group). The infarct area (white) from each section was measured (relative to LV area). Scale bar = 3 mm. B, Representative images of haematoxylin and eosin (H&E) were shown (n = 6 each group). Scale bar = 1 mm. C, Representative images of WGA staining were shown in the border zone of MI (n = 4 each group). Scale bar = 20 μm. D, Representative images of Masson staining were shown (n = 6 each group). Scale bar = 1 mm. E, Representative images of calculation of infarct size from each section of H&E staining (relative to LV area). F‐G, Representative images of calculation of myocyte size and myocyte number in the border size of MI based on WGA staining. H, Representative images of calculation of collagen volume fraction from each section of Masson staining (relative to LV area).Values expressed as mean ± SD; *P < .05, **P < .01, **P < .001

Ventricular remodelling following MI is characterized by cardiomyocyte loss, compensatory myocardial hypertrophy and cardiac fibrosis, resulting in changes in the size, shape and function of the heart.^19^, ^20^, ^21^ In the present study, the heart tissues from the lenti‐si lncRNA MEG3 group exhibited less spherical shape than those from the lenti‐GFP group at 4 weeks post‐LAD ligation in gross specimen (Figure 2A). Furthermore, H&E staining revealed a significant reduction in the infarct size of the mice with lenti‐si lncRNA MEG3 (Figure 2B,E).

Wheat germ agglutinin (WGA) staining was performed to show the contour and measure of cardiomyocyte size.^22^, ^23^ As shown in Figure 2C, the staining results indicated that the hearts from MI group exhibited enlarged cardiomyocyte size and reduced cardiomyocyte number in border zone compared with those of the sham group at 4 weeks post‐MI. The increased cardiomyocyte size and decreased cell number were reversed by treatment with lenti‐si lncRNA MEG3 (Figure 2C,F,G). However, no significant difference was noted in myocardial cell size and number in the remote zone between animals with lenti‐si lncRNA MEG3 and lenti‐GFP (Figure S2A‐C). We additionally performed Masson staining to analyse the extent of cardiac fibrosis. The results revealed a significant decrease in collagen volume fraction of the hearts from the lenti‐si lncRNA MEG3 mice compared with those of the lenti‐GFP group, indicating lower degree of cardiac fibrosis (Figure 2D,H).

Taken together, these results suggested that knockdown of lncRNA MEG3 could inhibit cardiomyocyte hypertrophy, cardiomyocyte loss and cardiac fibrosis in the border zone following MI.

### 3.4 Knockdown of lncRNA MEG3 inhibited apoptosis in vivo and in vitro

**Figure 3:**
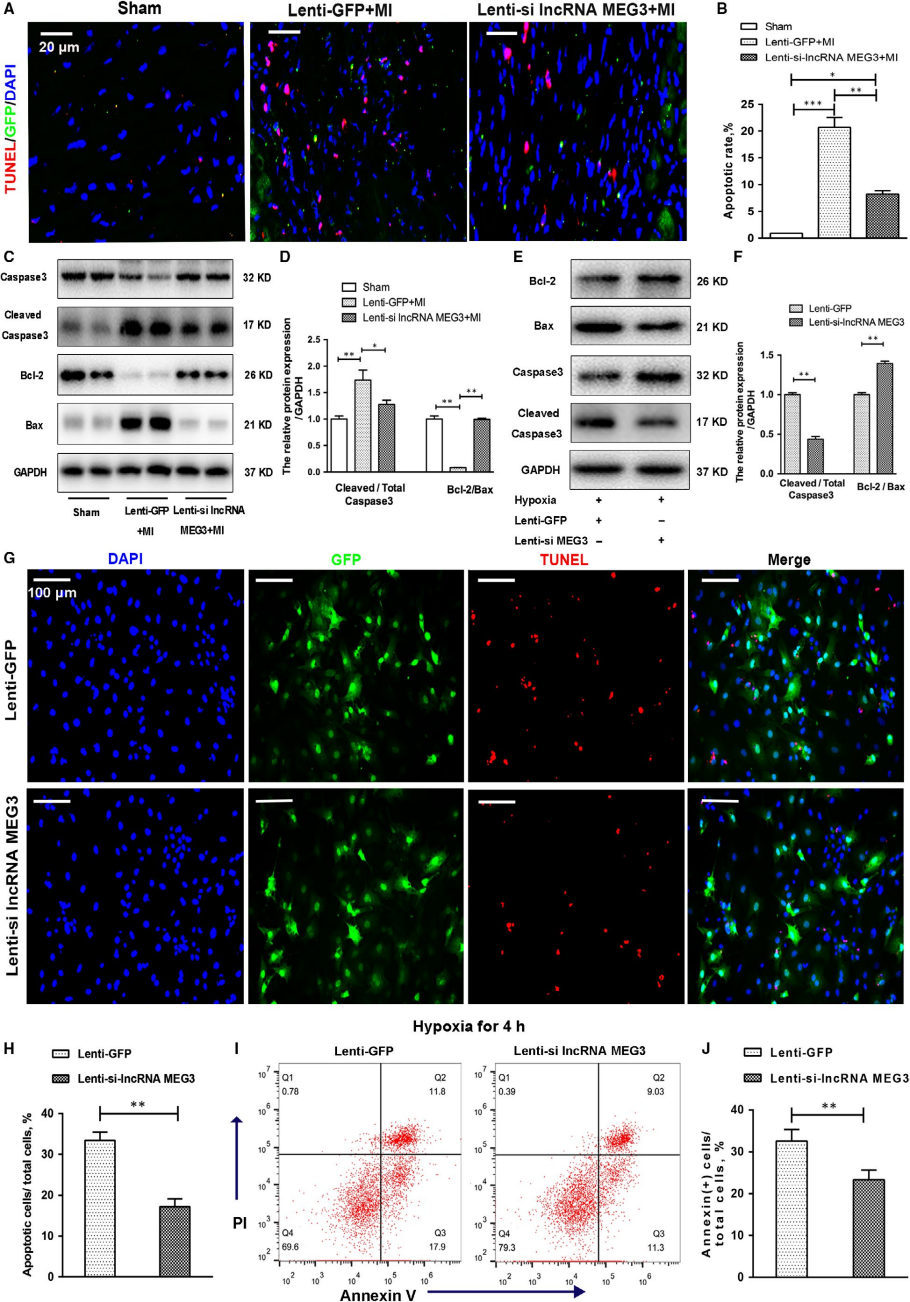
Knockdown of lncRNA MEG3 inhibited apoptosis in vivo and in vitro. A, Representative images of TUNEL staining showed cardiac apoptosis in infarct border zone from mice heart tissues. Scale bar = 20 μm. B, The apoptotic index was calculated by dividing the cell number of TUNEL‐positive nuclei by the total number of cells (n = 4 each group). C, Caspase 3, cleaved caspase 3, Bax and Bcl‐2 from mice heart tissues were detected by immunoblotting. The intensity of each band was quantified by densitometry, and the data were normalized to the GAPDH signal (D) (n = 7 each group). E‐F, Cardiomyocyte apoptosis was detected by flow cytometry 4 h after hypoxia. And the early apoptotic cells with Annexin (+) cells were calculated (n = 3 each group). G‐H, These protein expression levels of caspase 3, cleaved caspase 3, Bax and Bcl‐2 in hypoxia NMVMs were detected by immunoblotting and were quantified (n = 3 each group). I‐J, Representative images and quantification of cardiomyocyte apoptosis under hypoxia were detected by TUNEL staining (n = 3 each group). Scale bar = 100 μm. All data were reported as mean ± SD; *P < .05, ** *P* < .01, ***P < .001

It is well known that cardiomyocyte apoptosis is the predominant pathological phenotype in ventricular remodelling.^24^ In the present study, we performed terminal deoxynucleotidyl transferase dUTP nick end labelling (TUNEL) staining in order to evaluate cell apoptosis in the heart tissues of the animals following MI. A lower percentage of TUNEL‐positive cells was noted in lenti‐si lncRNA MEG3–treated groups compared with those noted in the lenti‐GFP–treated animals (Figure 3A,B), indicating the anti‐apoptotic effect caused by lncRNA MEG3 knockdown. The caspase 3 and Bcl‐2 protein family are the key molecules of the mitochondria pathway of apoptosis induction. Post‐MI induction, the levels of cleaved caspase 3 in infarct hearts exhibited a significant increase, whereas the ratio of Bcl‐2 to Bax was decreased, which could be reversed by MEG3 knockdown (Figure 3C,D). These results were also confirmed by immunohistochemical staining, which indicated decreased cleaved caspase 3 and increased ratio of Bcl‐2/Bax, supporting that lncRNA MEG3 knockdown attenuated myocardial apoptosis following MI (Figure S3A).

We further identified the anti‐apoptotic effect of lncRNA MEG3 on cultured cardiomyocytes under hypoxia. NMVMs were infected with lentivirus containing scramble NC and MEG3 shRNA‐1, and underwent hypoxia for 4 hours. Under hypoxic stress, lncRNA MEG3 knockdown significantly increased cell viability and decreased cell cytotoxicity in NMVMs (Figure S3B,C). No difference was observed between the MEG3 knockdown and the control groups under normal oxygen condition (Figure S3B,C). Similarly, the expression levels of caspase 3, Bcl‐2 and Bax were analysed by Western blotting. The levels of cleaved caspase 3 and Bcl‐2 were decreased and the levels of Bax were increased in lenti‐si lncRNA MEG3–treated NMVMs compared with lenti‐GFP–treated cells (Figure 3E,F). As showed in Figure 3G,H, the percentage of TUNEL‐positive NMVMs was reduced in lenti‐si lncRNA MEG3–treated compared with that noted in lenti‐GFP–treated NMVMs. In addition, the induction of apoptosis in NMVMs under hypoxic conditions was also assessed by flow cytometry using Annexin V/propidium iodide (PI) double staining. The percentage of Annexin (+) cardiomyocytes from the lenti‐si lncRNA MEG3–treated group was much lower than that of the lenti‐GFP–treated group after 4‐hour hypoxia (Figure 3I,J). These results indicated that lncRNA MEG3 knockdown protected cardiomyocytes against hypoxia‐induced apoptosis.

### 3.5 lncRNA MEG3 knockdown inhibited endoplasmic reticulum stress and oxidative stress

**Figure 4:**
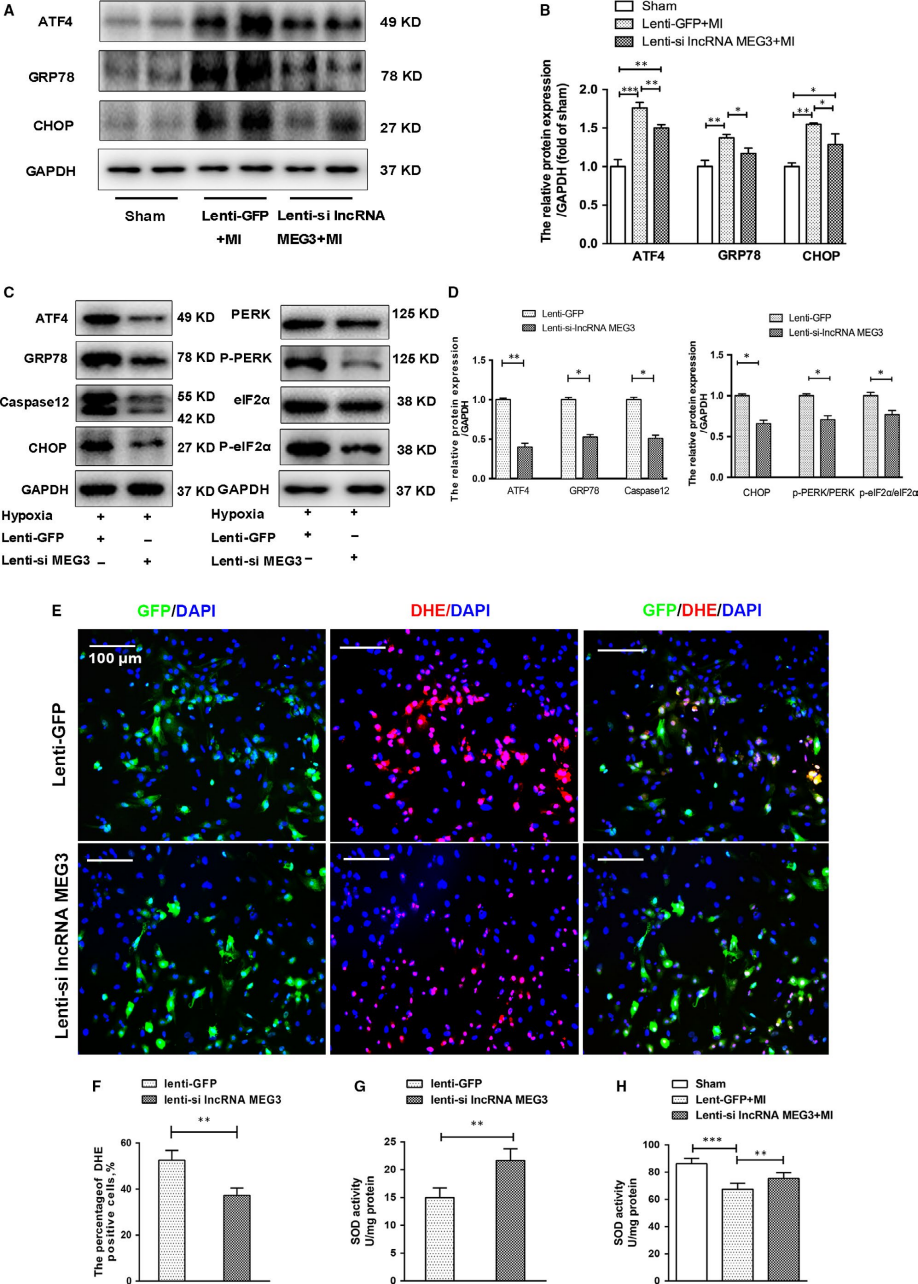
Knockdown of lncRNA MEG3 decreased ERS‐related protein expression levels and ROS production. A, Western blotting showed protein expression levels of GRP78, ATF4 and CHOP from mice hearts 4 wk post‐MI. B, The protein levels were quantified by densitometric analysis and normalized to the levels of GAPDH (n = 7 per group). C, Western blotting showed the expressions of ERS‐related proteins (GRP78, CHOP, caspase‐12, ATF4), and PERK‐eIF2α pathway (PERK, p‐PERK, eIF2α, p‐eIF2α) in hypoxia NMVMs. D, These protein expression levels were quantified and normalized to the levels of GAPDH (n = 3 each group). E‐F, Representative images and quantification of DHE staining were shown in hypoxia‐induced NMVMs in the absence or presence of lncRNA MEG3 knockdown. Scale bar = 100 μm. G‐H, SOD activities were detected in infarct cardiac tissues and hypoxic NMVMs after lentiviral transfection. Values expressed as mean ± SD; *P < .05, **P < .01

Endoplasmic reticulum stress (ERS) is triggered by ischaemia and can induce apoptosis in heart tissues.^4^ To elucidate whether inhibition of lncRNA MEG3 could protect against myocardial injury through the ERS pathway, we analysed the expression of the ERS‐associated proteins by Western blotting. In vivo, the expression levels of the myocardial ERS‐related protein markers, namely BIP/GRP78, ATF4 and C‐EBP homology protein (CHOP), were up‐regulated following MI. These effects were attenuated with the negative regulation of lncRNA MEG3 (Figure 4A,B).

Similarly, we demonstrated that the levels of GRP78, ATF4 and CHOP were down‐regulated in lenti‐si lncRNA MEG3–treated compared with lenti‐GFP–treated NMVMs under hypoxic conditions (Figure 4C,D). Previous studies have suggested that the PERK‐eIF2α and caspase 12 pathways were the main key players involved in ERS‐mediated apoptotic pathways.^4^, ^25^ In the present study, we observed the ratios of the expression of phosphorylation to total protein levels of PERK and eIF2α, and CHOP and caspase 12 were decreased under hypoxia in lenti‐si lncRNA MEG3–treated NMVMs compared with the corresponding levels in the lenti‐GFP–treated NMVMs (Figure 4C,D). It turned out that lncRNA MEG3 knockdown could inhibit ERS via the induction of PERK‐eIF2α and caspase 12 pathways post‐MI.

Previous studies have indicated that reactive oxygen species (ROS) are produced as a downstream event of ERS that can lead to cell death.^26^ We detected the ROS levels in hypoxic NMVMs by the dihydroethidium (DHE) assay. Pretreatment with lenti‐si lncRNA MEG3 reduced the increased number of DHE‐positive NMVMs that were induced by hypoxia, indicating lower ROS levels (Figure 4E,F). Superoxide dismutase (SOD) is an important antioxidant enzyme against oxidative damage. The SOD activity levels in lenti‐si lncRNA MEG3–treated NMVMs were significantly higher than those of the lenti‐GFP–treated cells (Figure 4G), as assessed by the SOD assay. In addition, the levels of SOD in heart tissues after MI were markedly decreased, and this effect could be increased in the absence of lncRNA MEG3 (Figure 4H). Taken together, the data suggested that lncRNA MEG3 knockdown could inhibit myocardial oxidative stress induced by hypoxia.

### 3.6 lncRNA MEG3 knockdown targeted and inhibited p53 protein

**Figure 5:**
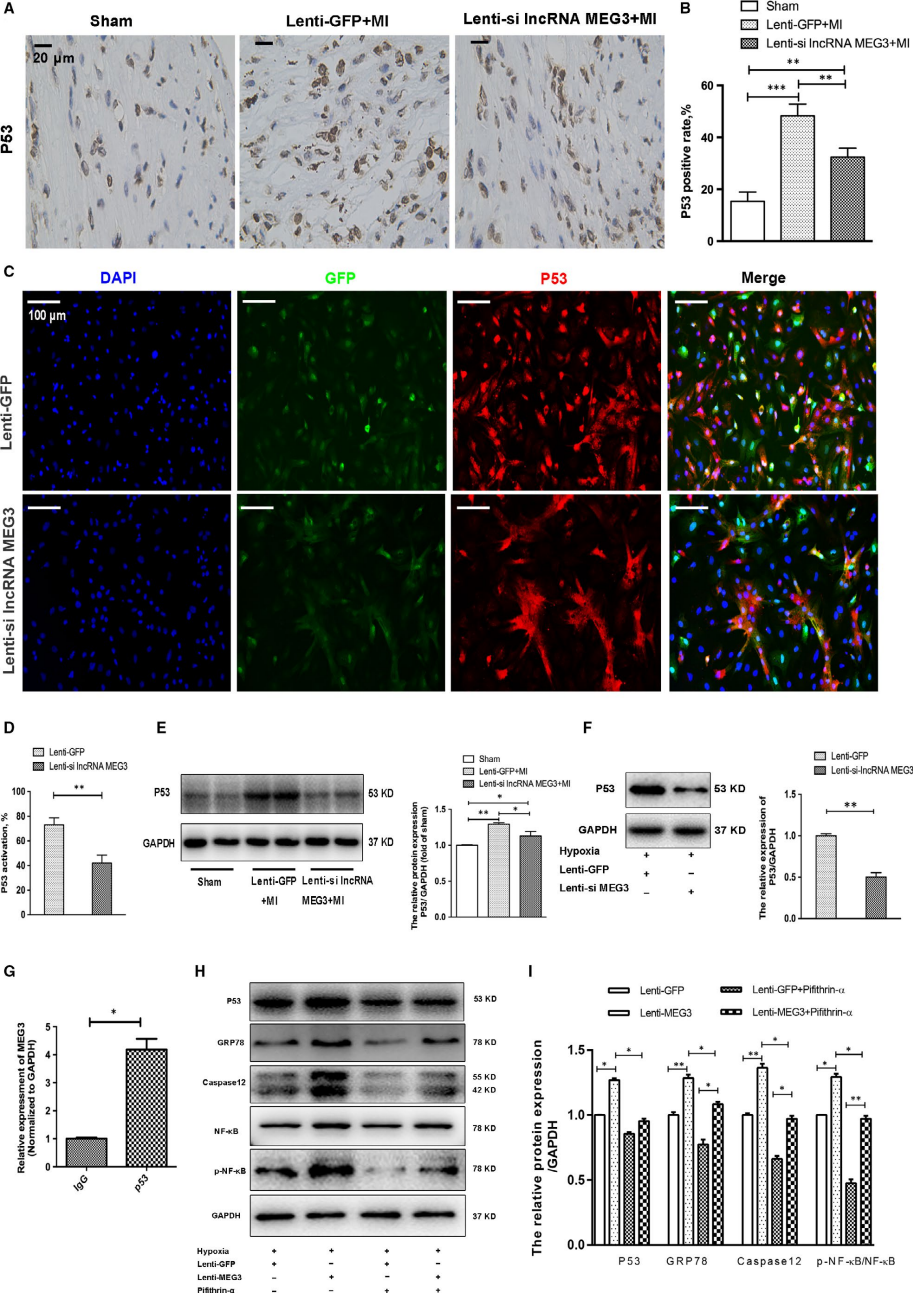
lncRNA MEG3 knockdown prevented cardiac injury by targeting and inhibiting p53 activation. A‐B, Representative images and quantification of immunohistochemical staining for p53 were shown in the border zone (n = 3 each group). Scale bar = 20 μm. C, P53 activation was shown by immunofluorescence in hypoxic NMVMs from two groups (n = 3 each group). Scale bar = 100 μm. D, Quantification of p53‐positive cells under hypoxia in each group. E‐F, The protein levels and quantification of p53 were analysed and normalized to the levels of GAPDH in infarct hearts (n = 7 each group) and hypoxic NMVMs (n = 3 each group). G, RIP experiments were performed with an antibody against p53 in extracts from hypoxic NMVMs. Purified RNA was used for qRT‐PCR, and the enrichment of the lncRNA MEG3 was normalized to GAPDH. The data were relative to mock‐IP (IgG). H‐I, The protein levels and quantification of p53, GRP78, caspase 12 p‐NF‐κB and NF‐κB were analysed and normalized to the levels of GAPDH in hypoxic NMVMs after lenti‐lncRNA MEG3 transfection with or without Pifithrin‐α treatment (n = 3 each group). All data were reported as mean ± SD. *P < .05, **P < .01

It has been shown that p53 is a crucial target of lncRNA MEG3 that affects cell proliferation and apoptosis.^11^, ^27^ We therefore explored the association of lncRNA MEG3 with p53 in infarct hearts and hypoxic NMVMs by immunochemical, immunofluorescence and Western blotting assays. The number of p53‐positive myocardial cells in the border zone was increased following MI, which could be inhibited by lncRNA MEG3 knockdown (Figure 5A,B). As shown in Figure 5C,D, p53‐positive cells were markedly decreased in NMVMs that were treated with lenti‐lncRNA MEG3 compared with those treated with lenti‐GFP vectors under hypoxic conditions. Moreover, Western blotting indicated that knockdown of lncRNA MEG3 decreased p53 expression compared with that noted in the control group in infarct myocardium and hypoxic NMVMs (Figure 5E,F). Besides, we also found that overexpression of lncRNA MEG3 in hypoxic NMVMs could result in increase in p53 and cleaved caspase 3, indicating a promotion for myocardial apoptosis (Figure S4A‐C).

In order to characterize whether p53 is a target of lncRNA MEG3, we detected the presence of lncRNA MEG3 in the p53 complex after precipitation with anti‐p53 antibody from NMVM extracts. RNA‐binding protein immunoprecipitation (RIP) assay demonstrated that lncRNA MEG3 could directly bind to p53 in primary NMVMs (Figure 5G). These results indicated that lncRNA MEG3 could directly interact with p53 and regulate p53 expression.

In order to further prove the functional link between ERS and p53, we detected the ERS‐related protein levels in hypoxic NMVMs transfected with MEG3 after treatment with Pifithrin‐α, a p53 inhibitor. In accord with a previous study,^28^ we found that inhibition of p53 reduced lncRNA MEG3 levels in hypoxic NMVMs (Figure S5A). Transfection with lenti‐MEG increased the levels of p53, GRP78 and caspase 12, which could be reduced by p53 inhibitor (Figure 5H,I). The results showed that ectopic expression of lncRNA MEG3 induced ERS‐mediated apoptosis through p53 pathway. Based on a previous study, the NF‐κB signalling might play a role in regulating p53‐related ERS.^29^ An increase in the ratio of p‐NF‐κB to NF‐κB levels was detected in the cells transfected with lenti‐MEG3, and this effect could be decreased in the inhibition of p53 by Pifithrin‐α (Figure 5H,I). Moreover, inhibition of ERS by 4‐PBA decreased the protein expression levels of CHOP and p‐NF‐κB (Figure S5B,C). These results demonstrated that lncRNA MEG3 might trigger NF‐κB and ERS‐related myocardial apoptosis through targeting p53.

## 4 DISCUSSION

In the present study, we initially observed that the expression levels of lncRNA MEG3 in ischaemic tissues and hypoxic NMVMs were increased. In addition, lncRNA MEG3 knockdown by lentivirus‐mediated delivery could reduce infarct size and cardiac fibrosis and improve cardiac function post‐infarction. Moreover, knockdown of lncRNA MEG3 decreased the induction of ERS‐related apoptosis and of oxidative stress in vitro and in vivo. Furthermore, we demonstrated that lncRNA MEG3 could target and regulate p53 protein levels in NMVMs. Finally, our data indicated that lncRNA MEG3 might induce NF‐κB‐ and ERS‐mediated myocardial apoptosis through p53 pathway.

The expression levels of lncRNA MEG3 were decreased by lentiviral delivery confirming that lncRNA MEG3 knockdown played a role in cardiac remodelling after MI. These improvements were observed in the EF and FS in lenti‐si lncRNA MEG3–treated animals by UCG, indicating improved cardiac function as a previous research showed.^28^ With regard to the parameters LVEDD and LVESD, a decline was noted at 1 week following MI, although the results were not statistically different most likely because of the relatively small number of animals used, which were not mentioned in Wu.et al research.^28^ Some reasons for the difference with our data were most likely because of different dose of lentiviral and longer time for hypoxia in cardiomyocytes, leading to no significant change in p53 levels. Beyond that, we also found that the improved cardiac function following lenti‐si lncRNA MEG3 treatment in mice was linked to the thicker scar and smaller infarct size. Moreover, we demonstrated that MEG3 knockdown could prevent cardiac fibrosis following 4 weeks of MI as described in a previous study with a transverse aortic constriction model.^30^ Hence, we confirmed that the knockdown of lncRNA MEG3 could prevent cardiac remodelling following MI. Myocardial apoptosis is mainly responsible for myocyte loss post‐MI and is considered the key link of ventricular remodelling.^31^, ^32^ We performed various techniques, such as TUNEL, flow cytometry, Western blot, CCK8 and LDH assays in order to demonstrate that inhibition of lncRNA MEG3 could protect against hypoxia‐induced apoptosis in cultured NMVMs and infarct hearts. In addition to apoptosis, we observed that lncRNA MEG3 knockdown could reduce ROS levels and increase SOD activity in hypoxic NMVMs and infarct myocardial tissues.

Current evidence has highlighted that lncRNA MEG3 was capable of targeting directly and activating p53 in various cell types.^11^, ^27^, ^33^ Consistent with these above reports, we identify that lncRNA MEG3 could directly bind to and regulate p53 expression in NMVMs by RIP and Western blot assays, but which were not entirely the same as Wu.et al research.^28^ Previous studies have found that p53 could also regulate the levels of GRP78, CHOP and Bax, which exerted key roles in the process of ERS, leading to apoptosis.^34^, ^35^, ^36^ However, the regulatory mechanism between p53 and ERS remains unclear. Studies indicate that NF‐κB signalling was reported to be a vital in regulating ERS‐ and p53‐related myocardial apoptosis.^29^, ^37^ In the present study, we observed that overexpression of lncRNA MEG3 significantly increased p53‐, NF‐κB‐ and ERS‐related protein levels, which were decreased after p53 inhibitor treatment. In addition, we also revealed that lncRNA MEG3 silencing abolished the increase in ERS‐related protein levels in vitro and in vivo*.* Collectedly, these findings clarified that lncRNA MEG3 knockdown could contribute to the prevention of NF‐κB‐ and ERS‐mediated myocardial apoptosis via targeting p53 protein.

Certain reports have proven that ERS plays an important role in the induction of myocardial apoptosis following MI.^38^, ^39^ Our data verified that ERS was activated undergoing MI, and was suppressed in the absence of lncRNA MEG3. The PERK‐eIF2α pathway interacting with ERS up‐regulates CHOP, which results in decreased expression of anti‐apoptotic protein Bcl‐2 and increased expression of pro‐apoptotic protein Bax, therefore activating cellular apoptosis.^40^ In addition, PERK‐eIF2α pathway can also activate caspase 12 and subsequently cleaved caspase 3, resulting in the induction of apoptosis.^41^, ^42^, ^43^ In the present study, we analysed the protein expression of ERS markers and of the Bcl‐2 family members using Western blotting in accord with previous studies.^44^ Besides, we also found inhibition of ERS by 4‐PBA could decrease NF‐κB expression levels. These data showed that lncRNA MEG3 knockdown could regulate myocardial ERS‐related apoptosis following MI via p53 and NF‐κB signalling.

Recent studies have suggested that ER signalling pathways correlate with ROS production.^26^, ^45^, ^46^ In response to ERS, the activation of CHOP was shown to promote ROS production and to indirectly disturb reduction–oxidation (redox) homeostasis causing further induction of oxidative stress.^47^, ^48^ In addition, ROS has also been reported to induce ERS that is involved in the CHOP‐Wnt pathway.^49^ As persistent ischaemia occurred, the ROS and ERS can eventually form a positive feedback loop, leading to further myocardium injury, such as induction of apoptosis. The present data have demonstrated that lncRNA MEG3 knockdown could suppress p53 expression and p53‐related ERS. Eventually, lncRNA MEG3 knockdown could disrupt the positive feedback loop between ROS and ERS.

The present study exhibits certain limitations. Firstly, it provides direct evidence that lncRNA MEG3 binds to p53 in murine cardiomyocytes. The concrete mechanism of lncRNA MEG3 on p53‐mediated ERS remains to be explored further, though the NF‐κB signalling has been represented. In addition, the gain‐of‐function approaches of lncRNA MEG3 should be carried out in vivo not only in vitro, which can further confirm its role in murine MI. Lentiviral vectors can cause stable transduction and long‐term transgene expression compared with adenoviral vectors.^50^, ^51^ However, their safety and efficiency require further investigation.

In conclusion, the current findings demonstrate that lncRNA MEG3 knockdown protect cardiomyocytes from the induction of apoptosis and ROS, and contribute to reduce cardiac remodelling and improvement of the cardiac function. P53‐related ERS and NF‐κB signalling pathways might be involved in lncRNA MEG3 knockdown–mediated therapeutic effect. Therefore, knockdown of lncRNA MEG3 might be a potential new target for the protection against ischaemic myocardial injury.

## CONFLICT OF INTEREST

The authors confirm that there are no conflicts of interest.

## AUTHOR CONTRIBUTIONS

Xueling Li, JinXuan Zhao, Jun Xie and Biao Xu conceived and designed the experiments; and Xueling Li, JinXuan Zhao, Jin Geng, Fu Chen, Zilun Wei, Chen Liu, Xinlin Zhang, Qiaoling Li, Jingmei Zhang, Ling Gao, Jun Xie and Biao Xu performed the experiments, analysed the data and wrote the manuscript.

## Data Availability

The raw data supporting the conclusion of this manuscript will be made available by the authors, without undue reservation, to any qualified researcher
