# Supplementary material for: Long non‐coding RNA MEG3 knockdown attenuates endoplasmic reticulum stress‐mediated apoptosis by targeting p53 following myocardial infarction
Source: J Cell Mol Med. 2019 Oct 20;23(12):8369–80. doi: 10.1111/jcmm.14714 (PMC6850962; doi:10.1111/jcmm.14714)
Supplement: Supplementary file 1 [file JCMM-23-8369-s001.doc]

**Supplementary materials**

**Data S1: Materials and methods**

***1.1 Lentiviral Transfection of Primary Neonatal Mice Ventricular Myocytes (NMVMs)***

The siRNA sequence that specifically targets MEG3 was inserted into the GV248 (pFU-GW-007-hU6-Ubiquitin-EGFP-IRES-puromycin) vector (Genechem, Shanghai, China), and a scramble sequence was used as a negative control. The siRNA sequences were as follows: MEG3 shRNA-1: 5′-GCTGCTTTCCTTCCTCACCTCCAAT-3′; MEG3 shRNA-2: 5′-CAGGACCCTCCAACTGTAAAT-3′; Scramble NC: 5′-TTCTCCGAACGTGTCACGT-3′. Neonatal Mice Ventricular Myocytes (NMVMs) were successfully infected with lentivirus as a multiplicity of infection (MOI) of 50. We further screened out the sequence with the highest inhibitory efficiency of lncRNA MEG3 in NMVMs by Taqman quantitative Real-Time PCR.

To further validate the relationship between lncRNA MEG3 and p53, the full length of MEG3 (Gen-BankTM NR_002766) with artificial EcoRI and AgeI enzyme restriction sites was cloned into the lentivirial expression vector. Successful cloning was confirmed by sequencing. We also accessed the successful overexpression of MEG3 in NMVMs after transfection by Taqman RT-PCR as shown in Fig.S4A.

***1.2 Quantitative Real-Time PCR***

Total RNA from heart tissues and cultured cells were isolated using the Trizol reagent (Invitrogen, Life Technology, USA). Total RNA was quantified using a NanoDrop 2000 spectrophotometer (NanoDrop, Wilmington, USA), and 1μg of total RNA from each sample was reverse transcribed into cDNA using HiScript II Q RT SuperMIx for qPCR (Vazyme, Nanjing, China). Quantitative real-time PCR (qRT-PCR) was performed for lncRNA MEG3 using AceQ qPCR Probe Master Mix Kit in a Stepone Plus Real-Time PCR system (Applied Biosystems) at 95°C for 30 second(s), followed by 45 cycles of 95°C for 10 s and 60°C for 30s. The qRT-PCR data were normalized to the average levels of the housekeeping gene GAPDH. Relative expression levels of mRNA levels were described as 2^−ΔΔCt^ value. The sequences of primers and the probes used for PCR are listed as follows: Forward: 5′- AACAAACAAGATGCTTACAGAA -3＇,Reverse: 5＇- GCACCCCGATACTAACCTA -3＇,Probe: 5＇-ATACGAGAGGTGTGAGCATGC- -3＇(lncRNA MEG3); Forward: 5＇- GAGAAACCTGCCAAGTATGATGAC-3＇, Reverse: 5＇- AGAGTGGGAGTTGCTGTTGAAG -3＇,Probe: 5-ATACGAGAGGTGTGAGCATGC- -3＇(GAPDH).

***1.3 Ultrasonic cardiogram (UCG)***

The mice cardiac function was evaluated by transthoracic UCG before, 1week and 4week after viral delivery. The mice were sedated with isoflurane and placed on a warmed platform. The mice hearts were scanned using M-mode and the echocardiographic parameters, such as left ventricular end-systolic diameter (LVESD) and left ventricular end-diastolic diameter (LVEDD) were measured with a Vevo2100 High-Resolution Micro-Ultrasound System (Visual Sonics, Toronto, Canada). Fractional shortening (FS) and ejection fraction (EF) of left ventricular diameters were calculated as described previously [1, 2].

***1.4 Western blotting analysis***

Western blotting was performed as previously described [3]. Adult male mice were sacrificed and their hearts were collected at 4 weeks following MI. Primary NMVMs were pretreatment managed for 24h using 0.5mm 4-PBA (sigma,USA) or 10μm Pifithrin-α (sigma,USA) after lentvirus transfection and hypoxia for 4h. Heart tissues and cells were lysed using RIPA protein extraction reagent with protease inhibitors (Beyotime, shanghai,China). Then equal amounts of proteins were loaded on 10-15% SDS-PAGE gels and blotted onto PVDF membranes. PVDF membranes were blocked with 5% non-fat dried milk in TBST and incubated with primary antibodies overnight at 4℃. The primary antibodies used in western blot analysis were antibodies against p53, Bcl-2, Bax, GRP78, ATF4, ANP, NF-κB (1:1000, abcam, UK), caspase3, PERK, P-PERK, eIF2α, P-eIF2α, ATF4, CHOP (1:1000, Cell signaling Technology, lnc.), and GAPDH (1:5000, Bioworld Technology, Inc.) as a loading control. After washing twice, membranes were incubated with appropriate HRP-conjugated secondary antibodies for 2 h at room temperature. Signals were detected using the ECL chromogenic substrate and quantified by densitometry by Quantity One software (Bio-Rad, Berkeley, CA).

***1.5 Apoptosis analysis***

Flow cytometric detection of apoptosis was performed with Annexin Apoptosis Detection Kit APC (eBioscience, lnc.) NMVMs infected with lenti-GFP or lenti-si lncRNA MEG3 were harvested by trypsinization at 72h after hypoxia, washed twice and suspended in 1x binding buffer. Following double staining with 5μl allophycocyanin (APC)-Annexin V and 10μl propidium iodide (PI) for 10 min at room temperature in the dark, then cells were examined by FACScan (BD Biosciences, California, USA) equipped with Cell Quest software (BD Biosciences).

A DeadEnd Fluorimetric TUNEL Kit (Roche,USA) was used evaluate cardiomyocyte apoptosis in infarct hearts and hypoxic myocardial cells. Heart tissues were fixed in 4% paraformaldehyde embedded in paraffin and sectioned at 5 µm intervals. Heart tissues sections and myocardial cells were fixed and permeabilized. Subsequently, they were incubated with the TUNEL reaction mixture that contained TdT and TMR-dUTP for 2 h at 37℃. After washing by PBS, the red labels in cellular nucleus were visualized by a Lecia fluorescence microscope (Leica, Germany). Then TUNEL-positive cells in total cells of overlapping filed were counted as the apoptotic ratio.

***1.6 Cell viability and*** ***cytotoxicity assay***

Cell viability was determined using the cell counting kit-8 (CCK8; Dojindo,WTS,Japan) as described previously[4]. Cells were seeded in 96-well plates at 5×10^3^cells/well. Cells were transfected with control, lenti-GFP and lenti-si lncRNA MEG3 for 72 h, and were cultured under hypoxic or normal oxygen conditions for 4 h. Then CCK8(10µl)was added to each well immediately and incubated for 2h at 37℃. The wells were read for OD at 570nm (A570) using a microplate spectrophotometer. Similarly, LDH activity in culture media was analyzed using an LDH release assay kit (Beyotime,shanghai, China). After lentiviral transfection and hypoxia, cell culture media were collected in 96-well plates and were added 10 µl LDH release kit to incubate for 4h at 37℃. The wells were read at 490nm. Results were expressed as the percentage of LDH leakage, which was the ratio of LDH activity in the media to total LDH activity.

***1.7 Oxidative stress analysis***

SOD activities in infarct heart tissues and hypoxic NMVMs were evaluated by total superoxide dismutase assay kit with NBT (Beyotime,shanghai, China) according to the manufacturer’s instructions as previously described[5]. Briefly, after homogenate and centrifugation(12,000g, 15min,4℃), the supernatant of heart tissues and NMVMs following lentiviral treatments were used for SOD activities (relative activities) determination. The absorbance of the samples was estimated at 560 nm using a microplate reader. SOD activity was expressed as units per milligram of protein.

Intracellular oxidative stress was determined using dihydroethidium (DHE) according to the manufacturer’s instructions as indicated previously [6]. NMVMs with lentiviral treatments were incubated with 10µM DHE(Beyotime, shanghai, China) for 30min at 37℃after hypoxia in the dark. The cells were observed under fluorescence microscope and quantified using the Image J software.

***1.8 Immunofluorescence***

Immunofluorescence staining was performed according to the protocol described previously [7]. The primary antibodies used were as follows: rabbit anti-p53 (1:200, abcam, UK), mouse anti-a-actin (1:200, abcam, UK). Each section was washed with PBS and incubated with cy3-labeled goat anti-rabbit or cy3-labeled goat anti-mouse lgG (H+L) (1:200, abcam, UK) at room temperature for 2h on the following day. DAPI was used for the nucleus staining. The cardiomyocyte size and the number of these cells in border zone of LV were assessed following staining with Wheat Germ Agglutinin (WGA) coupled with Alexa Fluor ^TM^ 594 conjugate (Invitrogen) as described previously[8]. Images were acquired using a Lecia fluorescence microscope (Leica, Germany).

***1.9 Histology and immunohistochemistry (IHC) analysis***

Heart tissues were fixed in 4% paraformaldehyde embedded in paraffin and sectioned at 5 µm intervals. Hematoxylin/eosin (H&E) and Masson’s trichrome were performed using standard procedures as described previously [9]. For IHC analysis, the sections were incubated with primary antibodies against caspase3, Bax, and Bcl-2 (1:100, abcam, UK). The slides were incubated with ABC kit and DAB substrate reagent (Vector Laboratories, USA). Then the slides were counterstained with haematoxylin and analyzed using the Image J software.

**References**

1. **Li, Q., J. Xie, R. Li, et al.**, Overexpression of microRNA-99a attenuates heart remodelling and improves cardiac performance after myocardial infarction*.* *J Cell Mol Med*, 2014. 18(5): p. 919-928.

2. **Perman, J.C., P. Bostrom, M. Lindbom, et al.**, The VLDL receptor promotes lipotoxicity and increases mortality in mice following an acute myocardial infarction*.* *J Clin Invest*, 2011. 121(7): p. 2625-2640.

3. **Wang, K., B. Long, L.Y. Zhou, et al.**, CARL lncRNA inhibits anoxia-induced mitochondrial fission and apoptosis in cardiomyocytes by impairing miR-539-dependent PHB2 downregulation*.* *Nat Commun*, 2014. 5: p. 3596.

4. **Sun, Z., X. Nie, S. Sun, et al.**, Long Non-Coding RNA MEG3 Downregulation Triggers Human Pulmonary Artery Smooth Muscle Cell Proliferation and Migration via the p53 Signaling Pathway*.* *Cell Physiol Biochem*, 2017. 42(6): p. 2569-2581.

5. **Xu, L., Y. Yu, R. Sang, et al.**, Protective Effects of Taraxasterol against Ethanol-Induced Liver Injury by Regulating CYP2E1/Nrf2/HO-1 and NF-kappaB Signaling Pathways in Mice*.* *Oxid Med Cell Longev*, 2018. 2018: p. 8284107.

6. **Qin, G., J. Xia, Y. Zhang, et al.**, Ambient fine particulate matter exposure induces reversible cardiac dysfunction and fibrosis in juvenile and older female mice*.* *Part Fibre Toxicol*, 2018. 15(1): p. 27.

7. **Piccoli, M.T., S. Gupta, J. Viereck, et al.**, Inhibition of the Cardiac Fibroblast-Enriched lncRNA Meg3 Prevents Cardiac Fibrosis and Diastolic Dysfunction*.* *Circ Res*, 2017.

8. **Mahmood, A. and L. Pulakat**, Differential Effects of beta-Blockers, Angiotensin II Receptor Blockers, and a Novel AT2R Agonist NP-6A4 on Stress Response of Nutrient-Starved Cardiovascular Cells*.* *PLoS One*, 2015. 10(12): p. e0144824.

9. **Li, X., J. Zhou and K. Huang**, Inhibition of the lncRNA Mirt1 Attenuates Acute Myocardial Infarction by Suppressing NF-kappaB Activation*.* *Cell Physiol Biochem*, 2017. 42(3): p. 1153-1164.

**Supplementary Figure**

**
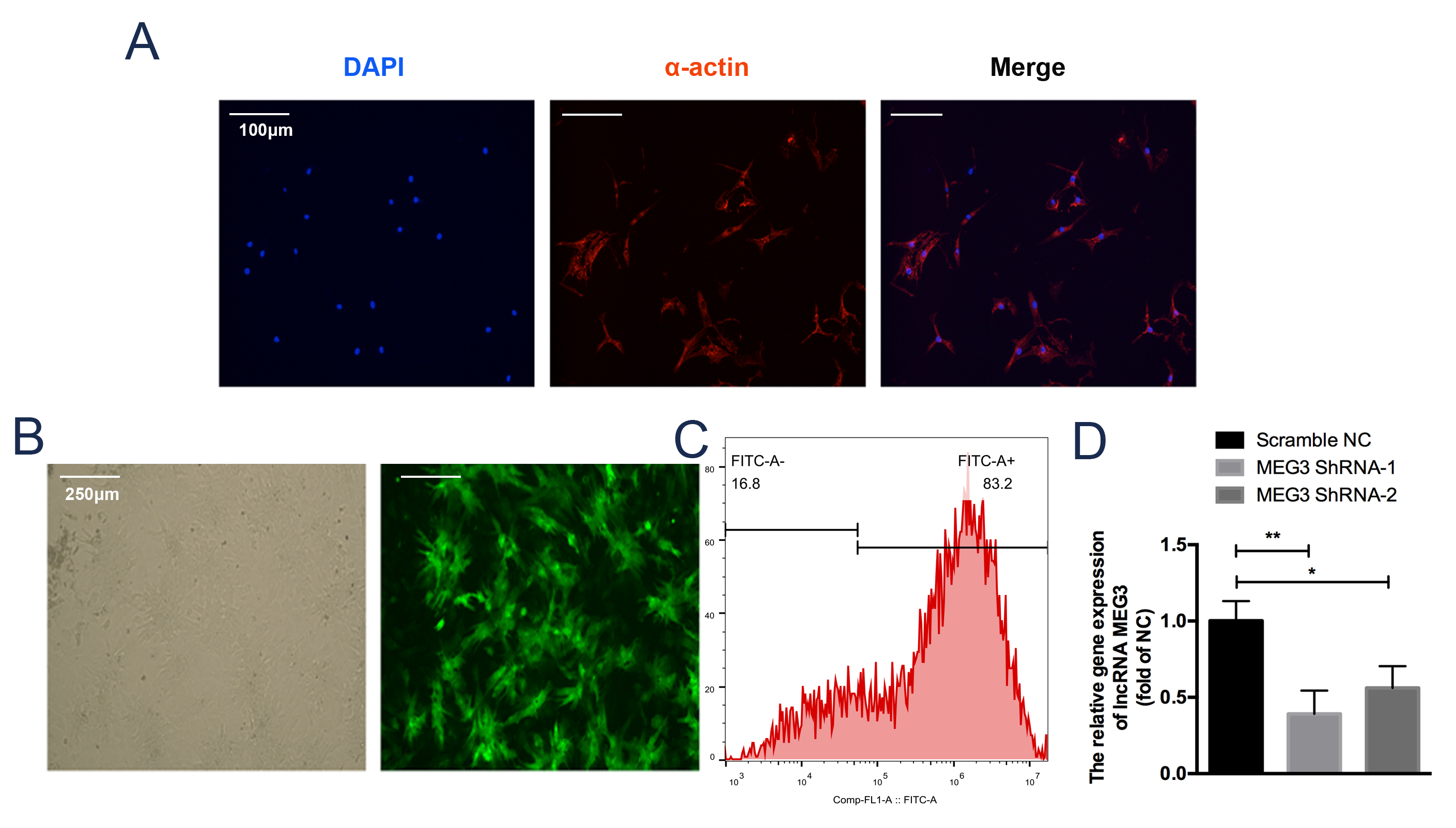
**

Fig. S1. Infection efficiency of cultured NMVMs. (A). Immunofluorescence staining of cultured myocytes usingα-sarcometric actin (Red) and DAPI (Blue). Scale bar=100μm. (B). Images of cultured NMVMs after lentivirus infection (MOI=50) for 72h in light microscope and Immunofluorescence staining of GFP. Scale bar=250μm. (C). Flow cytometry showed the percentage of cardiomyocytes with GFP after infection. (D). QRT-PCR showed the inhibition efficiency of lncRNA MEG3 in hypoxic NMVMs after transfection with MEG3 shRNA-1 and MEG3 shRNA-2 (n=3 each group). All data were reported as mean±SD. *P<0.05, **P<0.01.


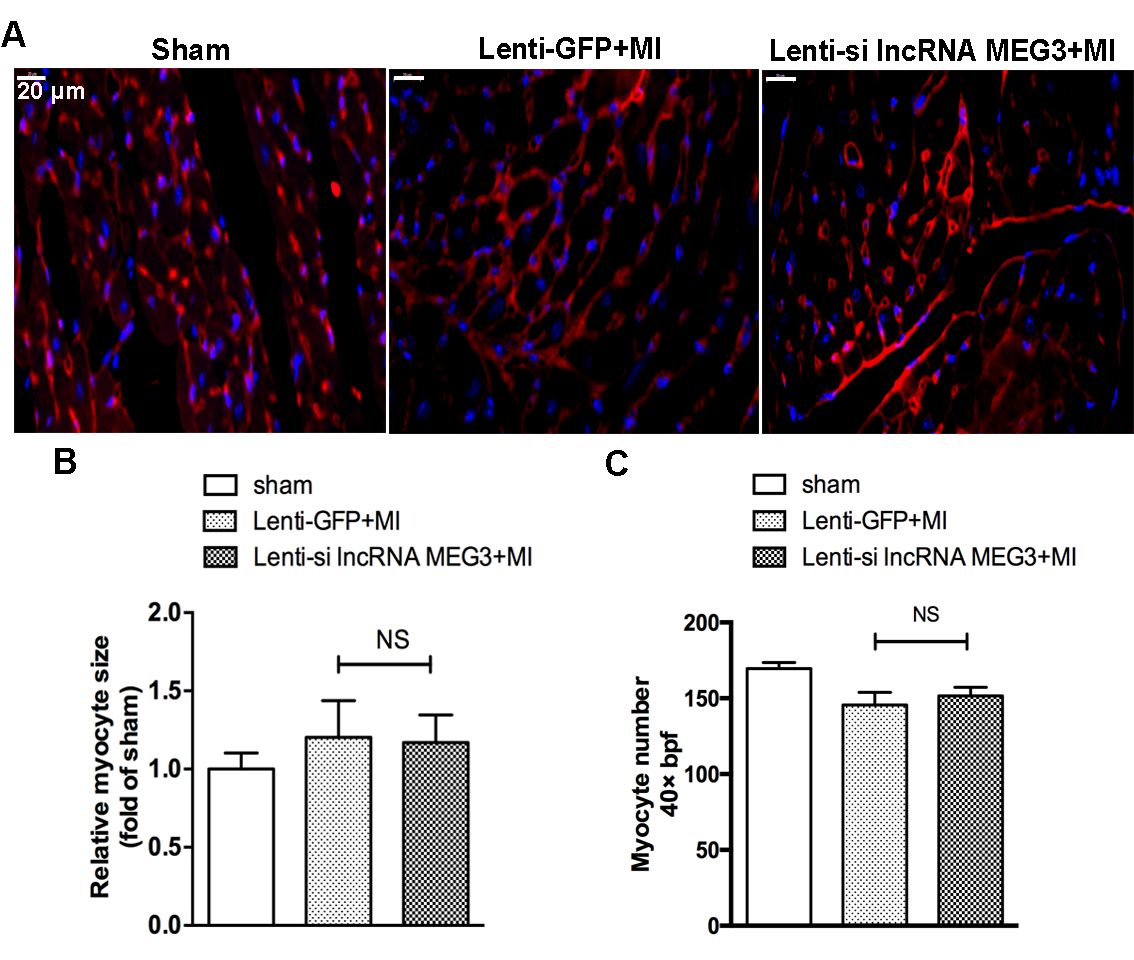


Fig. S2. (A). Representative images and quantification of WGA staining were shown in the remote zone of MI. Scale bar=20μm. (B-C). Quantification of myocytes size and number in the remote zone of MI were calculated by WGA staining (n=4 each group). NS: negative significance.


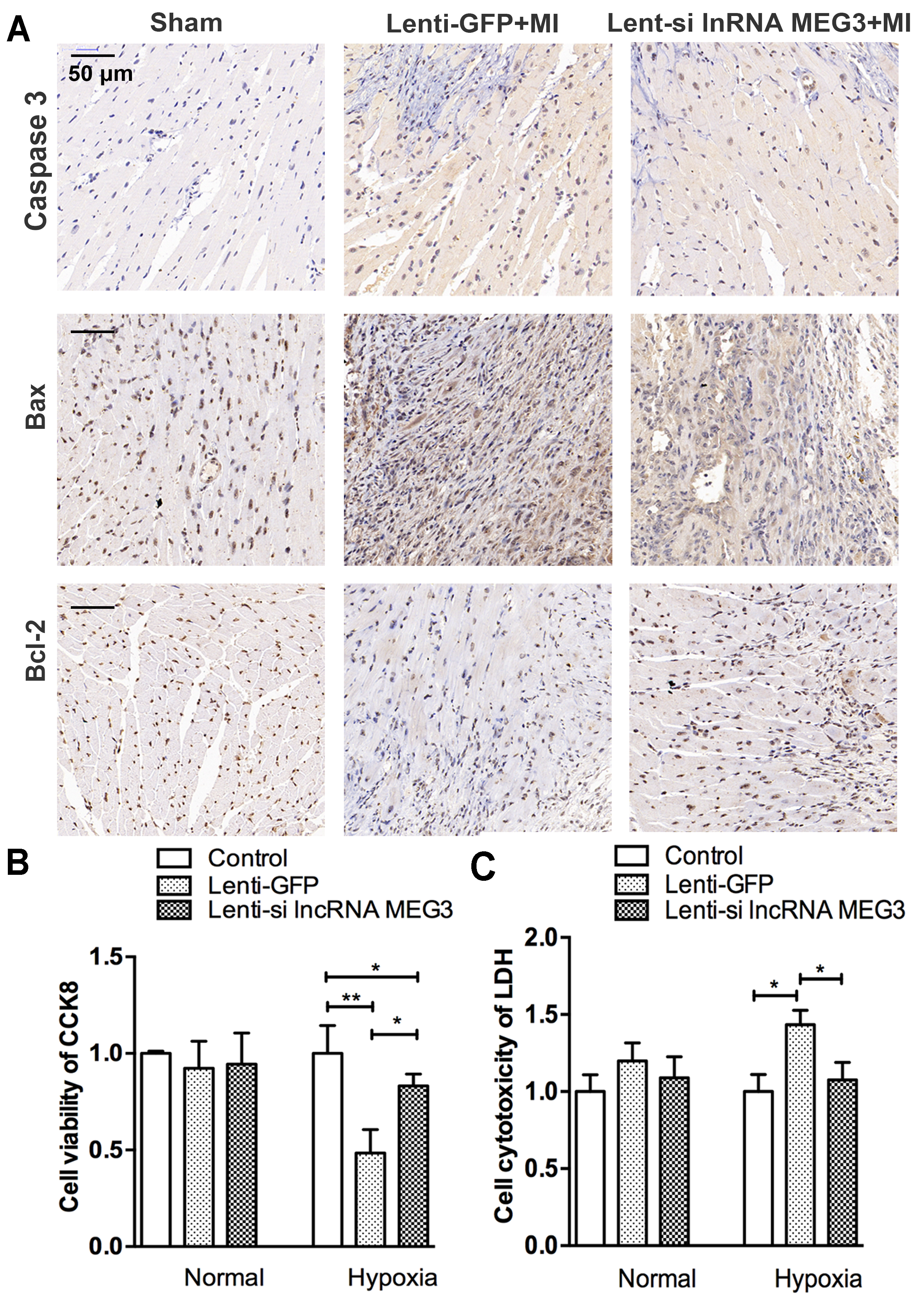


Fig. S3. (A). Representative images of immunohistochemical staining for Caspase3, Bax, and Bcl-2 in the border zone 28 days following MI (n=4 each group). Scale bar=50μm. (B). Cardiomyocytes viability after infection for 72h under normal oxygen and hypoxia were determined by the CCK8 assays (n=5 each group). (B). Cariomyocytes cytotoxicity were detected by LDH release assays under normal oxygen and hypoxic conditions (n=5 each group). All data were reported as mean±SD. *P<0.05, **P<0.01.


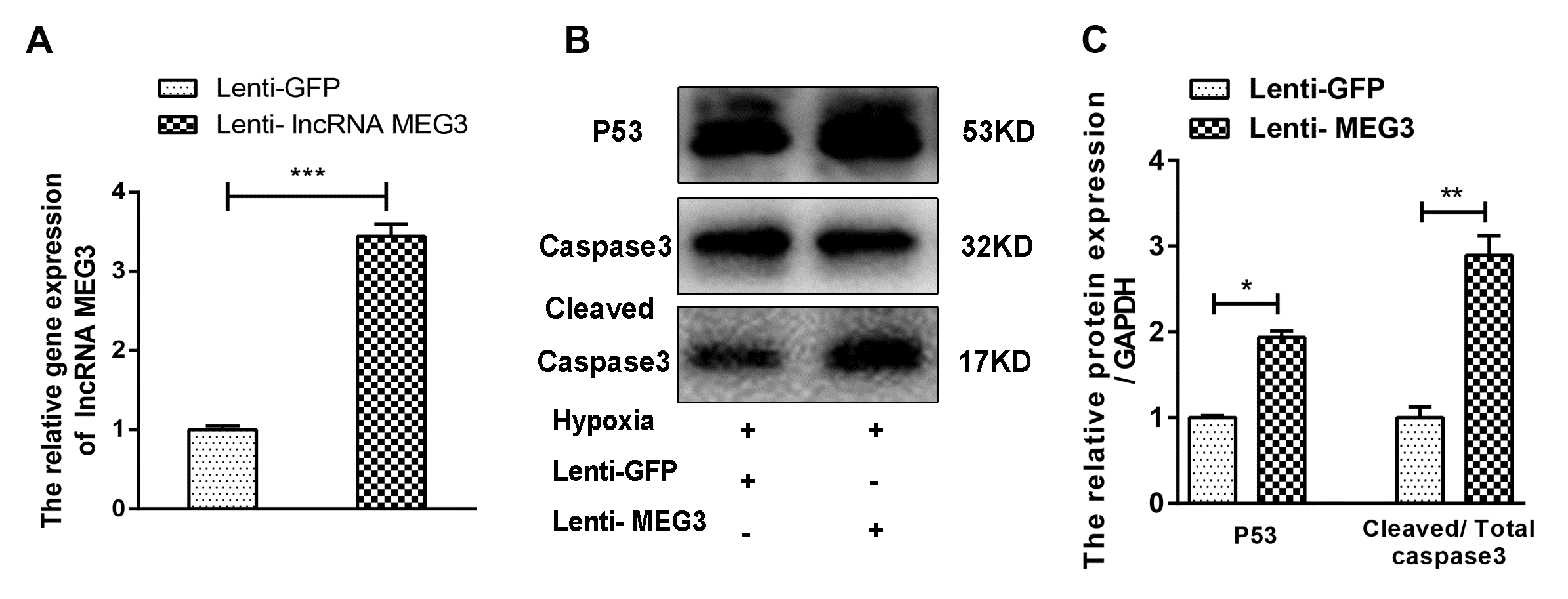


Fig. S4. (A). The qRT-PCR analysis of lncRNA MEG3 expression in hypoxic NMVMs after lenti-lncRNA MEG3 treatment (n=3 each group). (B-C). These protein expression levels of p53, Caspase 3 and cleaved Caspase 3 in hypoxic NMVMs were detected by immunoblotting and were quantified (n=3 each group).All data were reported as mean±SD. *P<0.05, **P<0.01.


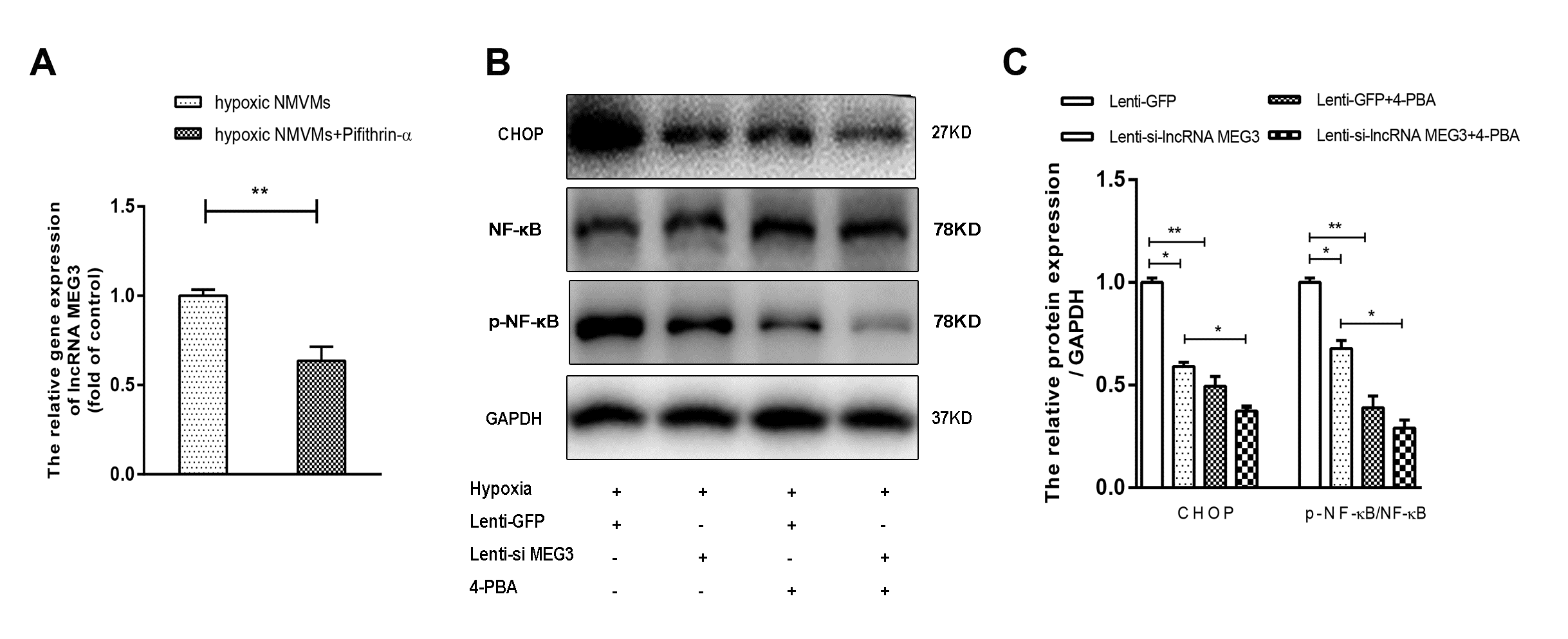


Fig. S5. (A). The relative gene expression of lncRNA MEG3 in hypoxic NMVMs after Pifithrin-α treatment (n=3 each group). (B-C). These protein expression levels of CHOP, p-NF-κB and NF-κB in hypoxic NMVMs with or without 4-PBA treatment were detected by immunoblotting and were quantified (n=3 each group).All data were reported as mean±SD. *P<0.05, **P<0.01.
